# Origin of nascent lineages and the mechanisms used to prime second-strand DNA synthesis in the R1 and R2 retrotransposons of *Drosophila*

**DOI:** 10.1186/gb-2009-10-5-r49

**Published:** 2009-05-05

**Authors:** Deborah E Stage, Thomas H Eickbush

**Affiliations:** 1 Biology Department, University of Rochester, 213 Hutchison, Rochester NY, 14627-0211, USA

## Abstract

**Background:**

Most arthropods contain R1 and R2 retrotransposons that specifically insert into the 28S rRNA genes. Here, the sequencing reads from 12 *Drosophila *genomes have been used to address two questions concerning these elements. First, to what extent is the evolution of these elements subject to the concerted evolution process that is responsible for sequence homogeneity among the different copies of rRNA genes? Second, how precise are the target DNA cleavages and priming of DNA synthesis used by these elements?

**Results:**

Most copies of R1 and R2 in each species were found to exhibit less than 0.2% sequence divergence. However, in many species evidence was obtained for the formation of distinct sublineages of elements, particularly in the case of R1. Analysis of the hundreds of R1 and R2 junctions with the 28S gene revealed that cleavage of the first DNA strand was precise both in location and the priming of reverse transcription. Cleavage of the second DNA strand was less precise within a species, differed between species, and gave rise to variable priming mechanisms for second strand synthesis.

**Conclusions:**

These findings suggest that the high sequence identity amongst R1 and R2 copies is because all copies are relatively new. However, each active element generates its own independent lineage that can eventually populate the locus. Independent lineages occur more often with R1, possibly because these elements contain their own promoter. Finally, both R1 and R2 use imprecise, rapidly evolving mechanisms to cleave the second strand and prime second strand synthesis.

## Abstract 2

Comparative analysis of 12 Drosophila genomes reveals insights into the evolution and mechanism of integration of R1 and R2 retrotransposons.

## Background

Transposable elements (TEs) are ubiquitous components and extensive manipulators of eukaryotic genomes. Because TEs constitute a significant mutation source and their remnants often comprise the majority of genomes, they are usually regarded as genomic parasites that are occasionally co-opted for host benefits [1,2]. While tracing the evolution of any genome should include a description of the natural history of its transposable elements, the diversity of TEs and their histories are so extensive that even with the advent of genome sequencing and assembly it remains challenging to follow the interplay between TEs and their host.

**Figure 1:**
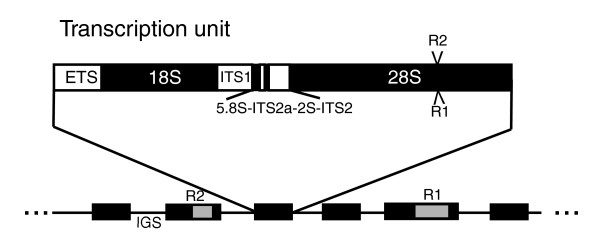
The rDNA loci of *Drosophila *species. Each rDNA transcription unit (diagramed in detail) consists of the 18S, 5.8S, 2S and 28S genes, the external transcribed spacer (ETS) and internal transcribed spacers (ITS1, ITS2a and ITS2). The location of the R1 and R2 insertion sites are indicated with arrowheads. Transcription units are separated by an internally repetitive intergenic spacer (IGS). The rDNA loci are usually, but not always, located on the X and Y chromosomes and typically contain hundreds of copies of the rDNA unit arranged in tandem arrays.

The rRNA genes provide a microcosm within the genome that is amenable to a detailed description of the interactions between TEs and their host. In eukaryotes these genes are organized into one or more loci, the rDNA loci, containing hundreds to thousands of copies of the 18S, 5.8S and 28S genes (Figure 1) [3]. A number of TEs specifically insert into the 28S genes of different animals [4]. The most extensively studied of these elements are the non-long terminal repeat (non-LTR) retrotransposable elements R1 and R2 of arthropods [5]. These two elements appear to have been inserting in the 28S genes of most arthropods since the origin of this phylum [6,7]. R2 elements have also been identified in a variety of other animal lineages [8,9]. The retrotransposition mechanism of R2 elements has been studied in detail [10,11]. The current model for their integration, called target primed reverse transcription (TPRT), has four basic steps: first, the bottom DNA strand of the target site is cleaved; second, the released 3' hydroxyl is used to prime cDNA synthesis by the element's reverse transcriptase; third, the top DNA strand is cleaved; and fourth, the released 3' hydroxyl is used to prime second-strand DNA synthesis [11]. This basic mechanism is likely used by R1 [12,13] and most other non-LTR retrotransposons [14].

Evolution of the rDNA locus is known to be dominated by concerted evolution, a recombinational process involving unequal crossovers and gene conversions that maintain near identity among repeats within a species while allowing those repeats to diverge between species [15]. Abundant evidence corroborates the extremely low sequence variation present among the many copies of the rDNA unit [16-18]. Sequence variants present at the lowest frequencies are equally distributed between the coding and non-coding regions of the unit. In contrast, the rare variants present at higher frequencies are greatly enriched in non-coding regions, indicating that selective pressures guide the extent of standing variation within the locus [18].

In arthropods from a few percent to over 50% of the rDNA units are inserted by R1 or R2 elements [19], and those units are thus prevented from producing functional 28S rRNA [20]. Within a species these many copies of R1 and R2 elements also exhibit low levels of sequence variation [21]. Surprisingly, divergent lineages of R1 or R2 are frequently found in a species, which cannot be explained by horizontal transfers between species [22]. This suggests that divergent lineages of elements must be able to form within a species.

The rDNA locus is not assembled as part of genome projects because of the highly repetitive nature of the rDNA locus. Thus, in this report we used the original sequencing reads generated from the 12 *Drosophila *genomes project [23] to address specific questions concerning the evolution and mechanism of integration of R1 and R2 elements. Can different lineages of R1 and R2 arise within a species despite concerted evolution maintaining sequence homogeneity among the rRNA genes? What is the location of second-strand DNA cleavage? How is this site used to prime second-strand synthesis in the retrotransposition reaction?

## Results and discussion

**Figure 2:**
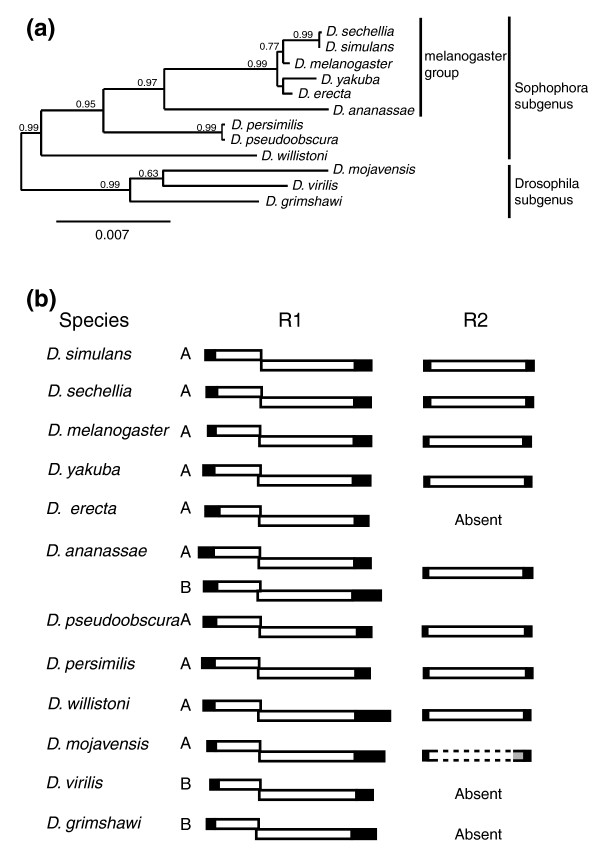
Phylogenetic relationships among the 12 sequenced *Drosophila *species and structures of R1 and R2 elements. **(a) **Phylogenetic relationships of the species based on maximum likelihood trees of their consensus 18S and 28S rRNA gene sequences. **(b) **Structures of the R1 and R2 elements found in each species. The 'A' and 'B' designations refer to the two divergent R1 lineages that are present among *Drosophila *species [28]. Filled rectangles correspond to the 5' and 3' untranslated regions (UTRs). Open rectangles correspond to the open reading frames (ORFs). R1 elements have two overlapping ORFs in different frames. *D. mojavensis *contains R2 elements but a complete sequence could not be assembled. No trace of R2 elements could be identified in *D. erecta*, *D. virilis *and *D. grimshawi*.

The phylogenetic relationships among the 12 *Drosophila *species used in this report are shown in Figure 2a. This phylogeny, based on the complete sequences of the18S and 28S genes, is consistent with the species relationships obtained with many other gene sequences [23]. In eight of the *Drosophila *species a complete R2 element could be assembled (Figure 2b; Additional data files 1, 2, 3, 4, 5, 6, 7 and 8). The structure of these elements conformed to previously identified R2 elements [24] and dN/dS analysis indicated that the assembled R2 elements had undergone purifying selection (mean dN/dS = 0.24 with a standard deviation of 0.321). In a ninth species, *D. mojavensis*, R2 sequences were identified but too few copies existed to assemble a complete sequence. R2 elements have been previously documented in several species groups of the *Drosophila *subgenus [25]; however, our failure to detect R2 sequences in *D. virilis *and *D. grimshawi *suggests R2 elements are frequently lost from this subgenus. The only example of R2 loss in the Sophophora subgenus, *D. erecta*, had been previously noted [26].

We also searched in all species for R2 copies that might be present outside the rDNA locus. We found no extra-rDNA R2 copies in *D. melanogaster*, as previously reported [27], or in *D. ananassae *or *D. persimilis*. *D. pseudoobscura*, *D. sechellia*, *D. simulans*, *D. willistoni*, and *D. yakuba *each had R2 copies not inserted in a 28S gene. These copies were frequently incomplete and all contained sequences that were from 1% to 2% divergent from those R2 copies within the rDNA locus. Thus, these non-rDNA copies of R2 could not have given rise to the current populations of R2 insertions in the rDNA locus. Finally, in *D. simulans *a fusion of the 5' end of an R1 element with the 3' end of an R2 element was identified as a tandem array outside the rDNA locus.

Complete R1 elements were assembled in all 12 sequenced genomes (Figure 2b; Additional data files 9, 10, 11, 12, 13, 14, 15, 16, 17, 18, 19, 20 and 21). The coding capacity of all R1 ORFs was consistent with previously characterized R1 elements [24]. A test of selection by dN/dS analysis indicated that the assembled R1 elements had undergone purifying selection (R1A, mean dN/dS = 0.30 with standard deviation of 0.376; R1B, mean dN/dS = 0.27 with standard deviation of 0.348). Previous analyses of R1 elements in *Drosophila *have suggested there are two distinct lineages of elements, A and B, that separated well before the origin of this genus and are differentially retained in the various species lineages [28]. Eleven of the sequenced *Drosophila *species contained a single R1 family of either the R1A or R1B lineage, while *D. ananassae *contained both lineages (Figure 2b). The only consistent difference in structure between the two lineages was that the two ORFs in the R1A lineage overlapped by 7 bp with a corresponding frame shift of -2, while the ORFs in the R1B lineage had a frameshift of -1 and overlapped from 14 bp in *D. ananassae *to 59 bp in *D. grimshawi*. As will be described below, in most species multiple examples were also identified of R1 insertions in non-28S gene locations.

### R1 and R2 intraspecies sequence variation

The average levels of sequence variation among the elements within each species are shown in Table 1. Because R1 insertions were found in genomic locations outside the 28S gene, we focused our analysis on the first and last 400 bp of each element and 100 bp of their flanking sequence to insure that all sequences were derived from copies located in the 28S rRNA genes. Except in the specific examples described below, the R1 and R2 elements in each species were extremely uniform, averaging less than 0.2% divergence from the consensus sequence. Because R2 elements are seldom present outside the locus, we also monitored nucleotide variation within internal regions of R2 elements in some species. Sequence divergence for central, coding regions of R2 were estimated at less than 0.1%, similar to or slightly lower than the 5' and 3' untranslated regions (UTRs; not shown).

**Table 1:** Variation in the 5' and 3' ends of R1 and R2 elements

	Major copy type: mean divergence* (maximum)		Atypical sequences: number (divergence)	
		
	5' end	3' end	Variant copies^†^	Variant 5' ends^‡^
**R1 elements**				
*D. simulans *R1A	0.000 (0.000)	0.002 (0.003)		4 (0.01-0.03)
*D. sechellia *R1A	<0.001 (0.003)	<0.001 (0.002)		
*D. melanogaster *R1A	0.001 (0.008)	0.003 (0.015)	2 (0.07)	
*D. yakuba *R1A	0.001 (0.005)	0.000 (0.000)	1 (0.02)	
*D. erecta *R1A	0.002 (0.005)	0.001 (0.007)		
*D. ananassae *R1A	0.013 (0.043)	<0.001 (0.003)		1 (0.08-0.11)
*D. ananassae *R1B	0.000 (0.000)	0.002 (0.005)		3 (0.15-0.22)
*D. pseudoobscura *R1A	0.000 (0.000)	0.002 (0.010)		1 (0.04)
*D. persimilis *R1A	ND	0.000 (0.000)	1 (0.02)	
*D. willistoni *R1A	0.002 (0.005)	0.001 (0.003)		
*D. mojavensis *R1A	0.000 (0.000)	0.000 (0.000)		
*D. virilis *R1B	0.001 (0.008)	<0.001 (0.003)	6 (0.01-0.05)	
*D. grimshawi *R1B_1_	0.000 (0.000)	0.001 (0.005)		
*D. grimshawi *R1B_2_	0.000 (0.000)	0.000 (0.000)		
				
**R2 elements**				
*D. simulans *R2	0.001 (0.008)	0.000 (0.000)		
*D. sechellia *R2	0.000 (0.000)	<0.001 (0.003)		
*D. melanogaster *R2	0.002 (0.005)	0.001 (0.015)	6 (0.02-0.05)	
*D. yakuba *R2	0.002 (0.013)	0.006 (0.018)		
*D. ananassae *R2	0.004 (0.008)	0.001 (0.005)		
*D. persimilis *R2	ND	ND		
*D. pseudoobscura *R2	ND	0.005 (0.010)		
*D. willistoni *R2 sub1	0.001 (0.003)	0.002 (0.008)		
*D. willistoni *R2 sub2^§^	0.003 (0.006)	0.015 (0.028)		

*Average nucleotide divergence from the consensus. The maximum divergence observed is shown in parentheses. ^†^Divergence detected at both the 5' and 3' ends except for the insertion in *D. yakuba*, where divergence was detected only at the 3' end. ^‡^The number of distinct 5' ends excludes the majority (consensus) sequence used in the previous columns. The divergence of additional 5' ends is calculated from the consensus. ^§^There may be only two copies of this lineage. ND indicates that these numbers were not included because sequencing reads with variant sequences had poor quality scores.

**Figure 3:**
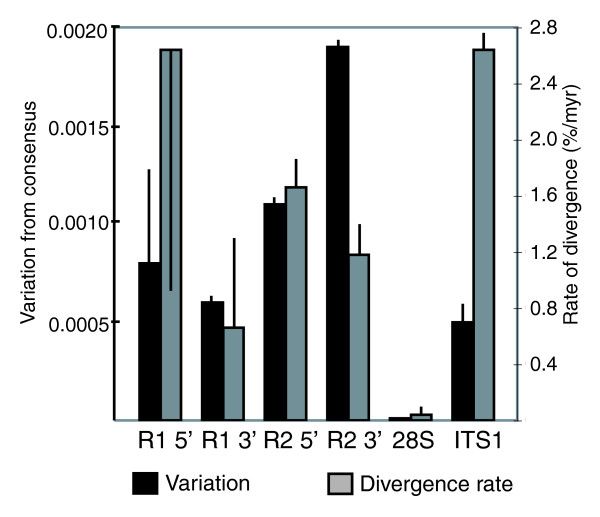
Average level of within-species sequence variation for R1 and R2. Sequence variation in the 400 bp at the 5' and 3' ends of the R1 and R2 elements from the 12 *Drosophila *species, the entire 28S rRNA gene and the internal transcribed spacer (ITS)1 region are shown (black bars). All values are calculated as the divergence from the consensus sequence for the species. Grey bars indicate the rates of nucleotide divergence (percent divergence per million years (myr)) of these same regions. Standard deviations are given for all values. The high standard deviation of the R1 5' end is a result of several species with no variation. The divergence data estimates were derived from comparison of the consensus sequences from *D. simulans*, *D. sechellia*, *D. melanogaster *and *D. yakuba *(divergence times: *simulans *versus *sechellia*, 0.25 myr; *simulans *or *sechellia *versus *melanogaster*, 3 myr; *simulans*, *sechellia *or *melanogaster *versus *yakuba*, 8 myr). Nucleotide variation data for 28S and ITS1 regions are derived from Supplemental Table 2 of [18].

In Figure 3 the level of nucleotide variation for the 5' and 3' ends of R1 and R2 shown in Table 1 are compared to the levels of nucleotide variation previously found in the 28S genes and internal transcribed spacer (ITS)1 regions of the rDNA units [18]. The levels of variation present in R1 and R2 were much higher than that of the 28S gene, and similar to that of the ITS1 region. We have previously shown that the level of nucleotide variation for different regions of the rDNA unit was proportional to the rate at which each region diverged between species [18]. This correlation is expected if all regions of the transcribed rDNA unit undergo similar levels of concerted evolution, because increased selective constraints on a sequence removes more variants that arise by mutation, which in turn enables fewer neutral variants to become fixed in all rDNA units (diverge over time). Also shown in Figure 3 (gray bars) are the nucleotide divergence rates of R1 and R2 compared to those for the 28S gene and the ITS1 region. These divergence rates were determined by comparing the consensus sequences of each region from *D. melanogaster*, *D. sechellia*, *D. simulans *and *D. yakuba*. The relationship between the levels of variation within a species and divergence rates between species that was observed for regions of the rDNA unit was not observed for the R1 and R2 sequences. For example, the 5' end of R1 evolved at four times the rate of the R1 3' end, yet had similar levels of nucleotide variation. The 5' and 3' ends of R2 evolved at one-half the rate of the ITS1 sequences, yet had two to four times the level of nucleotide variation within a species. In this latter example, the slower rate of divergence suggests that the R2 sequences are under greater selective pressure than the ITS1 sequences. Therefore, the finding that the R2 sequences have greater levels of variation suggests that they are not undergoing concerted evolution as effectively as the ITS1 sequences.

### Nascent subfamilies of R1 and R2

In addition to the many highly uniform copies of R1 and R2, five *Drosophila *species had one or more copies of R1 or R2 with nucleotide divergence of 1% to 7% from the consensus, clearly outside the range of divergences seen for the remaining R1 and R2 copies. The number and level of divergence of these atypical copies are listed in Table 1. Among these copies two had premature stop codons, indicating that they were inactive, while the remaining copies appeared to have intact ORFs. Because most divergent copies were not inserted into 28S genes that were also divergent, these R1 and R2 copies could represent distinct retrotranspositionally competent lineages of elements. However, the number of trace reads suggested these divergent R1s and R2s were at single copy levels, and thus it was likely that they had not recently been active.

Stronger evidence for the formation of nascent subfamilies was found in the examples of distinct 5' ends for the R1 elements of three species ('Variant 5' ends' column in Table 1). In *D. simulans *there were five distinct sequence classes of R1 5' ends, with each class representing from 11% to 26% of the total number of copies. There was no nucleotide divergence within each class, while divergence between classes ranged from 1% to 3%. In the case of *D. pseudoobscura *there were two distinct 5' ends. One-third of the R1 copies had 5' ends with over 4% nucleotide divergence from the remaining two-thirds of the R1 copies. Finally, R1 elements in *D. ananassae *showed the greatest tendency to diverge into subclasses with distinct 5' ends. The R1A elements could be separated into two classes that differed by 10% in nucleotide sequence, while the R1B elements could be separated into four classes that differed by 15% to 22% in sequence.

The separate lineages of the R1 5' ends observed in these three species were not apparent at the 3' ends of the R1 elements (that is, there was one class of 3' ends with mean levels of divergence less than 0.2%). Previous authors have suggested that new sublineages of transposable elements can arise within the same species by the acquisition of new promoter sequences [29,30]. Thus, one possibility is that the different 5' ends of R1 elements in a species correspond to rapidly evolving promoter sequences. R1 elements have been suggested to contain their own promoters because in some insect lineages R1 inserts in the opposite orientation in the 28S gene, or even outside the rDNA locus [9,31]. R2 elements on the other hand appear to be co-transcribed with the 28S gene and thus do not have their own promoter [32,33]. No evidence of divergent 5' ends was found for the R2 elements of any *Drosophila *species.

**Figure 4:**
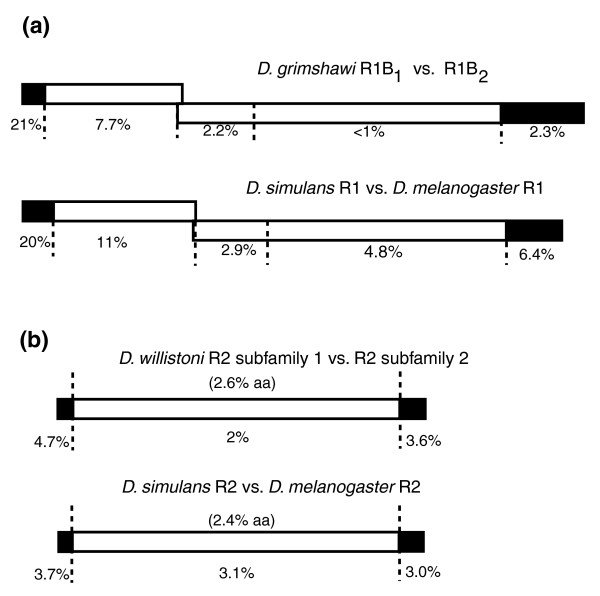
Summary of the nascent lineages of R1 and R2 in two species. In both panels the elements are diagramed as in Figure 2b. Values below the element diagrams are nucleotide divergences, while values above the diagrams are amino acid (aa) divergences. **(a) **Nascent lineages of R1 elements in *D. grimshawi*. For comparison, the relative level of sequence divergence between the R1 elements of *D. melanogaster *and *D. simulans *are also shown. The estimated time of separation of these two species is 3 million years [34]. **(b) **Nascent lineages of R2 elements in *D. willistoni*. The divergence between the R2 elements of *D. melanogaster *and *D. simulans *are shown.

Finally, Figure 4 summarizes two examples where the formation of nascent families involves sequence divergence of the entire R1 and R2 elements. In *D. grimshawi *two equally represented groups of R1B elements were detected that had 21% nucleotide divergence at their 5' ends and lower levels in other regions of the element (Figure 4a; Additional data files 12 and 22). The level of divergence for most regions of the two families was less than the divergence between the R1 elements of *D. melanogaster *and *D. simulans*, also shown in Figure 4a, suggesting the two R1B subfamilies in *D. grimshawi *are not as old as the estimated 3 million year separation between *D. melanogaster *and *D. simulans *[34]. The 5' ends of the subfamilies have undergone accelerated rates of divergence, similar to that described for the different 5' ends of R1 elements in *D. ananassae*, *D. simulans *and *D. pseudoobscura *(Table 1).

A second example of the presence of subfamilies within a species was found for the R2 elements in *D. willistoni*. In this case one subfamily, R2.1, was highly abundant while the R2.2 subfamily (Additional data file 23) was present in only a few copies. As shown in Figure 4b the subfamilies have diverged by 4.7% in their 5' UTRs and 3.6% in their 3' UTRs, similar to the divergence between the R2 elements of *D. melanogaster *and *D. simulans*. The amino acid divergence of the ORF from the two subfamilies (2.6%) was also similar to the divergence between *D. simulans *and *D. melanogaster *(2.4%), suggesting the divergence time between the *D. willistoni *subfamilies is similar to the time of divergence of *D. melanogaster *and *D. simulans*.

Unequal crossover events occurring within R1 (or R2) elements would homogenize their sequences, thus preventing the separation of two distinct lineages. Because the nucleotide divergence for most of the region encoding ORF2 of the R1B.1 and R1B.2 elements in *D. grimshawi *was less than 1%, and thus could still undergo recombination, we looked for evidence of such events. Blast searches were conducted using a query from the end of each subfamily. We then examined the sequence trace from the other end of each approximately 3.5 kb plasmid to determine whether it contained sequence from the same or opposite subfamily. Of 115 informative plasmid ends examined, only one pair indicated recombination between the subfamilies. This paucity of recombination can explain how these nascent subfamilies are able to avoid concerted evolution and remain independent lineages.

### Mechanism of R2 retrotransposition

#### Analysis of R2 junctions

**Figure 5:**
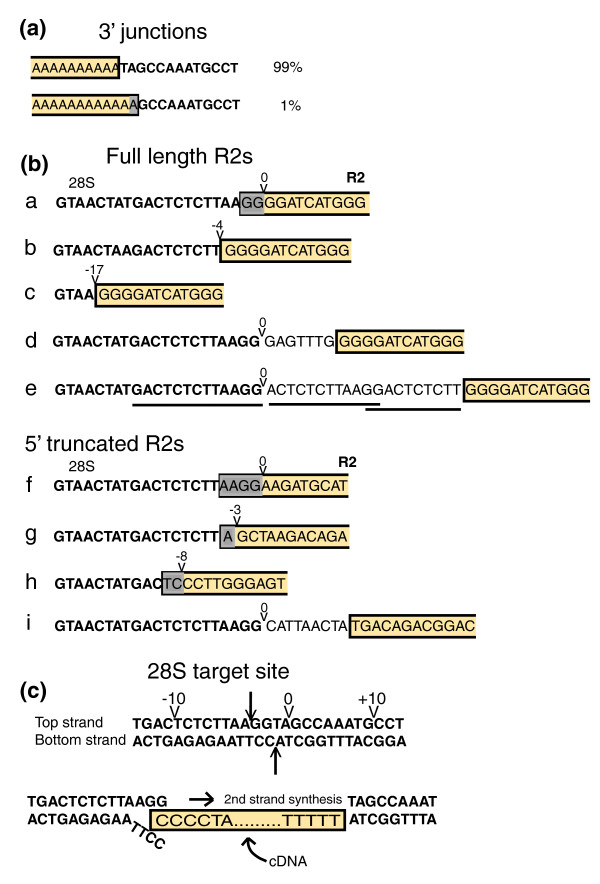
Junction sequences of the R2 elements with the 28S gene. **(a) **3' junction sequences. All *Drosophila *R2 elements contain 3' poly(A) tails. Most R2 insertions are consistent with the location of the R2 DNA cleavage sites on the bottom strand (see panel (c)) and its use in priming reverse transcription. **(b) **Representative examples of the 5' junctions of R2 elements with the 28S gene. All full-length examples are from *D. melanogaster*. R2 sequences are boxed, 28S sequences are in bold, non-templated sequences are in plain text, and duplications of 28S sequences are underlined. Boxed residues shaded grey correspond to microhomologies: sequences that could correspond to either the 28S sequence or the R2 element. **(c) **Location of the probable cleavage sites on the 28S gene. Arrows show cleavage locations determined *in vitro *for the R2 endonuclease from *B. mori *[10]; the arrow head topped by '0' shows the location of the top-strand cleavage site inferred after analysis of the *Drosophila *R2 5' junctions. The bottom diagram shows a hypothetical intermediate in the integration reaction after first-strand synthesis (boxed nucleotides) and second strand cleavage. The terminal two nucleotides of the cDNA are proposed to anneal to the top strand of the cleaved target site. This microhomology allows precise priming of second-strand DNA synthesis and the generation of the precise junctions seen in example a in panel (b).

As shown in Figure 5a, when viewed from their 3' junctions with the 28S gene, all R2 copies present in the sequenced *Drosophila *genomes were inserted into the same site as previously characterized R2 elements in all animals [5,35]. This location corresponds to the site of bottom strand DNA cleavage by the R2 endonuclease from *Bombyx mori *(Figure 5c). This cleavage site serves as the primer for reverse transcription of the element RNA [10]. For about 1% of the R2 insertions identified in the *Drosophila *genomes, bottom-strand cleavage appears to have occurred 1 or 2 bp downstream of this usual site. The uncertainty in cleavage location is because the second nucleotide downstream of the typical cleavage site is an 'A' and all *Drosophila *R2s end in a variable length poly-A tail (Figure 5a).

*In vitro *studies with the *B. mori *R2 endonuclease suggested the location of top-strand cleavage occurred 2 bp upstream of the bottom-strand site (Figure 5c) [10]. Previous analyses of a few R2 5' junctions from each of several *Drosophila *species, as well as other insect species, were interpreted in a manner that was consistent with such a cleavage [36,37]. However, there is significant variation at the 5' junctions of R2 elements, and the comprehensive analysis of this variation made possible using the genomic sequences suggested a reevaluation of this second-strand cleavage location was needed.

Similar to most non-LTR retrotransposons, R2 insertions can be full-length or contain truncations of their 5' ends. The 5' truncations have been suggested to be due to the failure of the reverse transcriptase to copy the entire RNA template, degradation of the RNA template, or the initiation of second-strand synthesis before reverse transcription is completed [5]. Figure 5b shows representative examples of full-length and 5'-truncated R2 elements. All full-length examples are from *D. melanogaster *but are representative of the R2 elements observed in all *Drosophila *species. Almost two-thirds of the full-length insertions have 5' junctions that include the 28S sequence to the position across from the site of bottom-strand cleavage, position '0' (examples a, d and e), with the remaining third containing variable deletions of the upstream 28S sequence (examples b and c). Many junctions contain additional bases at the junction. In some cases the additional bases represented duplications of the 28S gene (example e), while for other junctions the origin of the additional bases could not be identified, here called non-templated bases (example d). In the case of the 5' truncated elements most junctions contain from one to five bases at the precise 28S/R2 junction that may be assigned to either 28S or R2 (examples of these microhomologies are indicated by the shaded bases in Figure 5). R2 5' truncated junctions can also be associated with deletions of upstream 28S sequences (examples g and h), and non-templated additions (example i).

**Figure 6:**
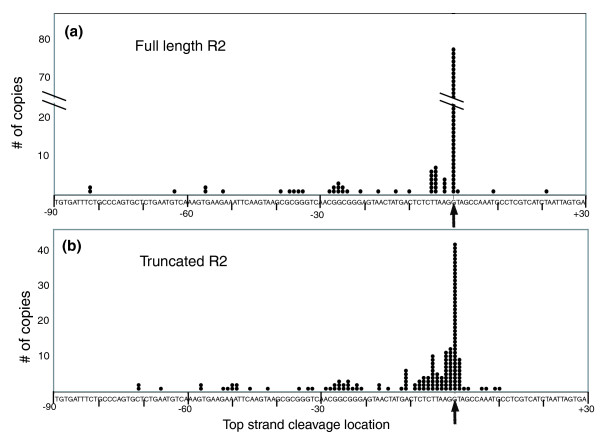
Probable top-strand cleavage sites for the R2 element insertions. Dots indicate for all R2 elements the last nucleotide at the 5' junction that corresponds to the upstream 28S sequence. In instances where multiple copies of an element within a species had identical junctions, the number of genomic copies was estimated by dividing the number of traces by the fold coverage of the genome sequencing project. Arrows show the location of the insertion site (bottom-strand cleavage used for TPRT). The data were obtained from the following species: *D. ananassae*, *D. melanogaster*, *D. pseudoobscura*, *D. sechellia*, *D. simulans *and *D. yakuba*. **(a) **Full-length R2 elements. **(b) **5' truncated R2 elements.

Figure 6 is an attempt to summarize the 5' junction data for the R2 copies in several species. Plotted in these figures is the last contiguous nucleotide of the 28S gene found for each R2 insertion. For most full-length copies the last 28S nucleotide corresponded to the position opposite the bottom-strand cleavage site (Figures 5c and 6a). In the case of the 5' truncated R2 elements (Figure 6b), more copies are associated with deletions of 28S sequences, but again the most frequent final base of the 28S gene is opposite bottom-strand cleavage.

#### R2 retrotransposition model

This analysis of the 5' junctions of R2 insertions supports the following additions to the TPRT model for R2 retrotransposition. First, the most frequently used top-strand cleavage site in *Drosophila *is directly opposite the bottom-strand site, rather than 2 bp upstream (position -2) as suggested from the *in vitro *studies with the R2 endonuclease from *B. mori *[10]. Cleavage opposite the bottom-strand site readily explains junctions such as examples d, e and i in Figure 5b, junctions difficult to explain if top-strand cleavage was at position -2. Second, we suggest that full-length R2 RNA transcripts in *Drosophila *contain G residues at their 5' end. All integrated full-length R2 copies in *D. melanogaster *begin with four G residues (examples a to e in Figure 5b), and similar analysis of the other *Drosophila *species indicated that the full-length R2 elements in these species contained at least two terminal G residues (data not shown). No conservation of R2 5' end sequences is found beyond these two Gs.

To explain the most frequently observed R2 full length junctions (example a in Figure 5b), we suggest that two terminal C residues on the cDNA strand made from the R2 transcript anneal to the cleaved target site (see Figure 5c for a diagram). A tendency to anneal a few nucleotides of the cDNA strand to the top strand of the target DNA before initiating second-strand DNA synthesis would explain the frequent 'microhomologies' between internal R2 sequences and upstream 28S sequences that are found associated with 5' truncated R2 insertions. The non-templated nucleotides found at the 5' junctions of full-length and truncated copies of R2 are suggested to result when the annealing of microhomologies does not occur. *In vitro *studies have shown that the R2 polymerase adds non-templated nucleotides before initiating from a primer that is not annealed to the template [38], as well as when it 'runs-off' the end of a template [39]. These non-templated nucleotides can be of any sequence and thus could also lead to microhomologies used to initiate polymerization of the top DNA strand. Microhomologies between these non-templated nucleotides and the 28S gene would go undetected by our analysis. Finally, the R2 junctions with deletions of the 28S gene, as well as the few with duplications of the 28S gene, could represent top-strand cleavages outside the preferred site.

### Mechanism of R1 retrotransposition

#### R1 junction sequences

**Figure 7:**
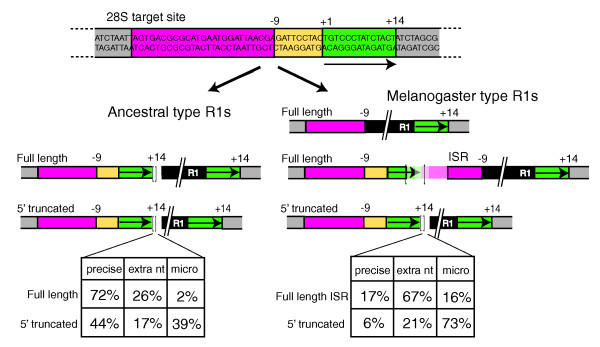
Full length and 5' truncated junctions of the *Drosophila *R1 insertions. Shown at the top is the sequence of the 28S gene insertion site. Various regions of the sequence have been indicated with colors to allow the 5' and 3' junctions of the R1 insertions to be summarized. Position +1 corresponds to the position of bottom-strand cleavage based on the 3' junctions of all R1 elements as well as from *in vitro *studies of the R1 endonuclease [12,13]. Positions -9 and +14 correspond to the inferred most frequent sites of top-strand cleavage. Shown at the bottom are diagrams of the 5' and 3' junctions of R1 insertions. Full-length as well as 5' truncated insertions of the ancestral type R1s have 14 bp target site duplications (left side). The bracketed region of the junctions exhibited sequence variation. This variation can correspond to non-templated nucleotides (sequences corresponding to neither the 28S gene nor the R1 element), or microhomologies (1 to 5 nucleotides that could correspond to either the 28S gene or the R1 element). Melanogaster group R1s have three classes of junctions (right side): full length insertions with a precise 9 bp target site deletion; full length insertions with an insertion site rearrangement (ISR); and 5' truncated insertions. Sequence variation at these junctions is limited to the bracketed region and corresponds to the variation seen in the ancestral type R1s. The tables at the bottom show the fraction of copies observed at the bracketed site that are precise, contain non-templated nucleotides (extra nt) or microhomologies (micro).

Based on their 3' junctions with the 28S gene, all R1 elements within the 28S gene are located 60 bp downstream of the R2 insertion site. *In vitro *studies with the *B. mori *R1 endonuclease suggest this site corresponds to the location of the bottom-strand DNA cleavage site used to prime a TPRT reaction [12,13]. As in the case of R2, the 5' ends of both full-length and truncated R1 copies showed significant variation (Figure 7).

Based on their 5' junctions the R1 elements could be divided into two groups. For R1B elements and the R1A elements outside the melanogaster species group, the upstream 28S gene sequences typically extended to a position 14 bp downstream of the bottom-strand site (+14), and thus a 14 bp target site duplication (TSD) flanks the R1 insertions (Figure 7, left side). Because similar length TSDs flank the R1 elements in most other arthropods [40,41], this group is called the 'ancestral type' R1 insertions. All sequence variation associated with the 5' junctions of both full-length and 5' truncated elements of the ancestral type were located at the end of the TSD (bracketed region in Figure 7). As with the R2 elements, these 5' junctions could be classified as precise, containing microhomologies, or containing non-templated nucleotides. Most full-length ancestral type R1 insertions were precise, with the remainder containing non-templated nucleotides. The 5' truncated ancestral R1s are more broadly distributed between precise, non-templated and microhomology junctions.

In contrast to the ancestral type R1s, many copies of the R1A elements in the melanogaster species group (*D. ananassae*, *D. erecta*, *D. melanogaster*, *D. sechellia*, *D. simulans*, and *D. yakuba*) contained upstream 28S gene sequences that extended only to position -9. The relative proportion varied from species to species, but altogether 75% of the full-length 'melanogaster-type' R1 insertions contained this 9 bp deletion. No 5' sequence variation was associated with these insertions. The remaining full-length insertions contained variable-length TSDs up to 17 bp in length and an unusual duplication of 28S sequences that we called insertion site rearrangements (ISRs). These duplications of the 28S sequence extended for 16 to 27 nucleotides upstream of position -9. Microhomologies and non-templated nucleotides were common at these junctions and were always located between the TSD and the ISR (bracketed region). The 5' truncated insertions of the melanogaster-type R1s also contained precise, non-templated and microhomology junctions.

**Figure 8:**
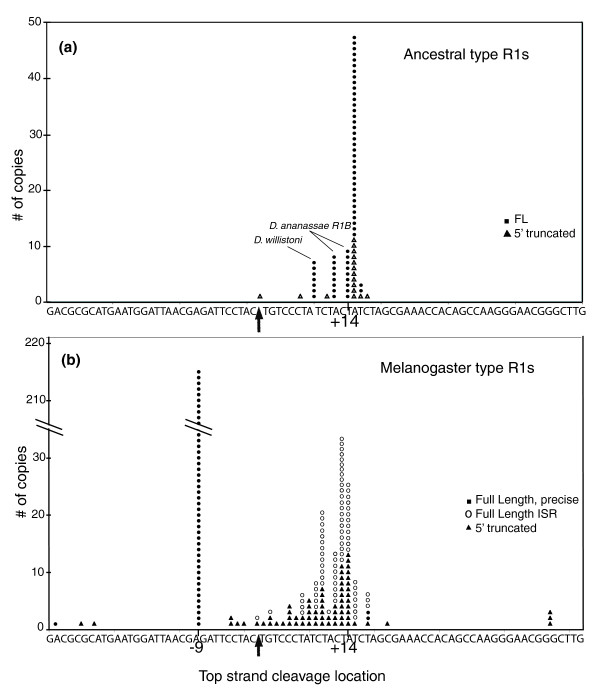
Probable top-strand cleavage sites for the R1 element insertions. Dots indicate for all R1 elements the last nucleotide at the 5' junction that corresponds to the upstream 28S sequence. In instances where multiple copies of an element within a species had identical junctions, the number of genomic copies was estimated by dividing the number of traces by the fold coverage of the genome sequencing project. Arrows show the location of the insertion site (bottom-strand cleavage used for TPRT). The different types of junctions diagrammed in Figure 7 are given different symbols. **(a) **Ancestral type R1 elements. Data derived from *D. ananassae A*, *D. mojavensis*, *D. pseudoobscua *and *D. willistoni*. **(b) **Melanogaster-type R1 elements. Data derived from *D. melanogaster*, *D. sechellia*, *D. simulans *and *D. yakuba*.

Figure 8 is again a plot of the last contiguous nucleotide of the 28S gene found at the 5' end of the R1 elements in several species. In the case of the ancestral type R1s, the full-length and 5' truncated junctions are consistent with a top-strand cleavage at position +14. Most exceptions to this cleavage location were in *D. ananassae*, where R1B insertions made an 11 or 13 bp TSD, and in *D. willistoni *where some R1B insertions contained an 8 bp TSD. The probable locations of the top-strand cleavage for the melanogaster-type full-length R1 elements (Figure 8b) were clustered about two locations. Precise full-length insertions had top-strand cleavage at position -9. For the full-length ISR elements and the 5' truncated elements top-strand cleavages were less specific but clustered around position +14.

#### Tandem R1 arrays

In seven of the *Drosophila *species R1 elements were found organized as tandem arrays (Table 2). Evaluation of the sequencing reads at the other end of clones containing tandem R1s revealed that many, and perhaps all, of these tandem R1 elements were located within the rDNA loci. These tandem arrays were located at the normal R1 insertion site with the individual R1 copies separated by the 14 bp 28S gene sequence corresponding to the typical TSD. Such R1 tandem arrays have been previously described in *D. virilis *[42]. Because R1 insertions were never found inserted upstream of R1 insertions without the TSD in these species, the mechanism of tandem R1 formation appears to be the insertion of additional R1 elements into the TSD present at the 5' end of R1 elements already inserted into a 28S gene. Consistent with this, melanogaster-type R1 elements have no or few tandem R1 insertions (Table 2, but see the legend). The highest levels of tandem insertions were in *D. pseudoobscura *and *D. ananassae *R1B where each R1-inserted rDNA unit contained an average of two to three R1 elements.

**Table 2:** Ratio of R1 elements in the canonical 28S site, tandem arrays and non-28S locations

Species	Fraction of rDNA units	Tandem*	Non-28S*
*D. simulans*	0.08	-	+
*D. sechellia*	0.62	+	+
*D. melanogaster*	0.11	-^†^	+
*D. yakuba*	0.11	-	+
*D. erecta*	0.36	+	-
			
*D. ananassae *R1A	0.17	++^‡^	++
*D. ananassae *R1B	0.13	+++	+++
*D. pseudoobscura*	0.40	+++	+++
*D. persimilis*	0.26	+++	++
*D. willistoni*	0.10	+	++
			
*D. mojavensis*	0.68	+	+
*D. virilis*	0.75	++	+
*D. grimshawi*	0.20	+	+

*Plus signs indicate: +, less than 0.4 of those in 28S; ++, from 0.4 to 0.9 of those in 28S; +++, as many or more than those in the 28S; -, indicates absence. ^†^Unlike the tandem R1s in the ancestral group, the sequence between the many tandem-like, consecutive R1 elements in *D. melanogaster *is typical of that found in an insertion site rearrangement (ISR) junction. Unlike the highly variable ISR junctions found in the locus, the ISRs between these tandem-like R1s are the same. Thus, these elements are likely the result of amplification of an original unique event. ^‡^All found upstream of R1B.

#### R1 insertions into non-28S locations

In most *Drosophila *species copies of R1 were also identified that had inserted into sequences outside the 28S gene (Table 2). Frequent R1 insertions outside the 28S gene have also been reported in *B. mori *[43]. The *Drosophila *species with the most abundant examples of non-28S R1 insertions were *D. ananassae *and *D. pseudoobscura*, the two species with the highest levels of tandem duplications. This may suggest that R1 insertions in these species are either less specific or retrotranspose more often. We identified 118 unique examples of non-28S gene R1 insertions that had intact 3' junctions, suggesting that they represented authentic retrotransposition events, not segments of R1 sequence that had been displaced by recombination to locations outside the rDNA locus. The insertion sites for these copies frequently corresponded to repeated DNA sequences, probably in the pericentromeric or telomeric regions of the genome. An exception was in *D. ananassae*, where a 22 bp region of the 28S gene corresponding to the R1 28S insertion site was found in the IGS region of the rDNA unit. One R1A element and seven R1B elements were found in rDNA units containing this unusual insertion of 28S sequences within the IGS.

**Figure 9:**
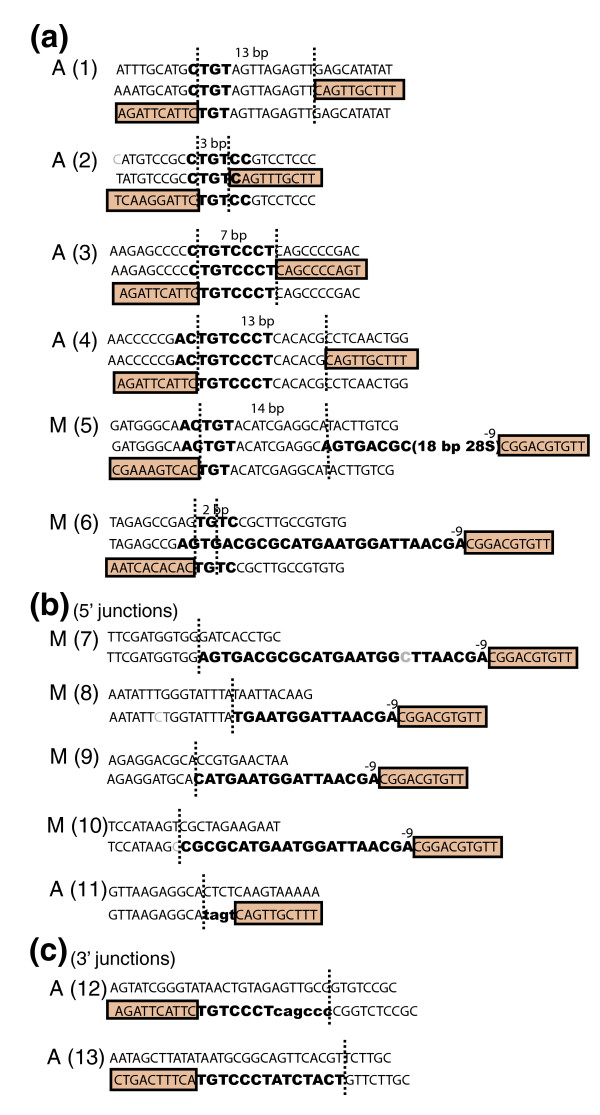
Junction sequences and the probable target sites of R1 elements inserted outside the 28S genes. The inferred uninserted target sequence is shown at the top of each example. Boxed sequences indicate R1, bold uppercase sequences are identical to 28S sequences, and bold lower case nucleotides are non-templated nucleotides. **(a) **Insertion sites in which both 5' and 3' junctions of the R1 element were recovered; 2 to14 bp target site duplications were created by the insertions and are delineated by vertical dotted lines. **(b) **Insertion sites in which only the 5' junction with R1 was recovered. **(c) **Insertion sites in which only the 3' junction with R1 was recovered. Junctions come from the following species: *D. pseudoobscura*, examples 1, 4 and 11; *D. mojavensis*, example 2; *D. persimilis*, examples 3 and 12; *D. sechellia*, examples 5, 8, 9 and 10; *D. yakuba*, example 6; *D. ananassae A*, example 7; *D. virilis*, example 13. M, melanogaster-type R1 elements; A, ancestral type R1 elements.

Because the insertions had occurred into repeated DNA sequences we were able to identify the likely target sequences for some of the R1 insertions. Figure 9 shows examples in which we were able to identify either or both 5' and 3' ends of the insertions and their likely target sequences. In all cases where both junctions were recovered (Figure 9a), the target sites contained from 4 to 9 bp of sequence identity to the 28S target site centered near the lower strand cleavage site, and the insertions generated TSDs of from 3 to 14 bp.

The 5' junctions of all melanogaster-type full length R1s outside the rDNA array contained upstream 28S gene sequences ending at position -9 and extending upstream for 7 to 28 bp (Figure 9, examples 5 to 10). The 5' ends of the ancestral type R1 insertions showed no evidence for the insertion of flanking 28S rRNA sequence (examples 1 to 4 and 11). The 3' junctions of both the ancestral and melanogaster-type R1s were similar, with most beginning at the precise 3' junction of the R1. However, some insertions showed the incorporation of up to 14 nucleotides of downstream 28S sequences (example 13) or the presence of non-templated nucleotides (example 12).

#### R1 retotransposition model

The analysis of the R1 5' junctions with the 28S genes, as well as the insertions into non-28S gene locations, enables us to propose several steps involved in the R1 retrotransposition reaction. These suggestions are based on the standard TPRT mechanism of R2 and other non-LTR retrotransposons and the ability of their polymerases to add non-templated nucleotides at the ends of synthesized DNA strands. First, because no variation was detected within a species or between species for the location of the bottom-strand cleavage used to prime reverse transcription, like R2, this is the most conserved step in the retrotransposition reaction of R1. The analysis of R1 insertions outside the 28S gene site revealed that downstream 28S gene sequences are sometimes also inserted with the R1 element. In addition, many of these non-28S gene target sites contain bases corresponding to the 28S gene (examples 1 to 6 in Figure 9). Together these data suggest that the 3' end of the R1 transcript used for retrotransposition often contains flanking 28S sequences. These flanking 28S sequences may anneal to the bottom strand of the target site and thus account for why there is little sequence variation associated with the 3' junctions of R1.

**Figure 10:**
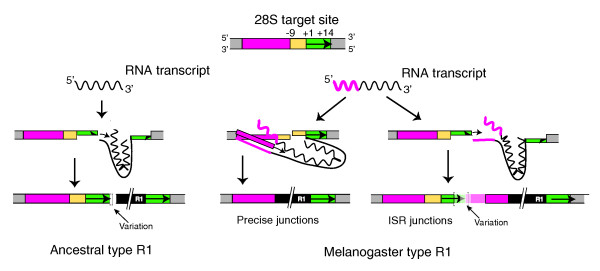
R1 retrotransposition models based on the standard target primed reverse transcription reaction. The uninserted 28S gene is shown at the top. The various regions upstream and downstream of the target site are colored as in Figure 7. Left side: ancestral type R1 transcripts (wavy line) do not contain upstream 28S gene sequences. Ancestral type R1s cleave the top DNA strand 14 bp downstream of the bottom cleavage site. Nucleotide variation at the 5' junctions corresponds to the imprecise nature by which the R1 polymerase uses the top strand of the DNA target to prime second-strand DNA synthesis. Right side: full length melanogaster-type R1 transcripts include 28S sequences starting upstream of position -9. Cleavage of the top strand occurs at one of two sites. If top-strand cleavage occurs 9 bp upstream of the bottom-strand site, then the upstream RNA sequences can anneal to the end of the cDNA strand, resulting in a precise 9 bp deletion of the target site. If top-strand cleavage occurs downstream of the bottom-strand site, then the annealing of cDNA to the target site is not possible, generating variation at the junction of the target site duplication.

For all ancestral type R1s, top strand DNA cleavage is predominantly located 14 bp downstream of the bottom-strand cleavage. This top-strand cleavage location, among others, was also detected for the R1 endonuclease of *B. mori *[12,13]. The use of this cleavage to prime second strand synthesis results in 14 bp target site duplications (Figure 10, left side). The only deviation in this 'ancestral' cleavage location appeared in *D. ananassae *R1B elements, which had 11 and 13 bp TSDs, and in *D willistoni *R1B elements where several examples of elements with 8 bp TSDs were detected. The priming of second-strand synthesis appears less precise than first-strand synthesis, sometimes involving the addition of non-templated nucleotides. This suggests that the cDNA strand does not efficiently anneal to the cleaved top strand, suggesting few if any nucleotides of upstream 28S sequence are included at the 5' end of the R1 transcript. Consistent with this suggestion, no flanking 28S sequences were associated with the ancestral-type non-28S R1 insertions.

R1A lineage elements in the melanogaster species group appear to have evolved changes in the TPRT reaction. In these R1s, top-strand cleavage frequently occurs 9 bp upstream of bottom-strand cleavage, resulting in a 9 bp target site deletion. Priming of second-strand synthesis from this site is highly precise, that is, non-templated sequences were never observed, suggesting 28S gene sequences are included at the 5' end of the RNA transcripts used for retrotransposition. This flanking 28S sequence allows the reverse transcribed strand to anneal to the target site (Figure 10, middle). Consistent with this suggestion, from 7 to 28 bp of flanking 28S sequences ending at position -9 are associated with the non-28S gene insertions of the melanogaster-type R1. The R1A elements in the melanogaster group also appear able to cleave the top DNA strand in a less specific manner around position +14 (Figure 8b). Such cleavages result in ISR junctions in which a typical TSD is present followed by 16 to 27 bp of the 28S gene ending at position -9 (Figure 10, right side). Considerable variation is detected in these junctions between the TSD and the duplicated upstream 28S sequences, presumably because this downstream cleavage eliminates the ability of cDNA sequences to anneal to the top strand in the initiation of second-strand synthesis. An intriguing aspect of these melanogaster-type R1 elements is that all 5' truncated insertions have TSDs (Figure 8), suggesting they can only arise from downstream cleavage of the top strand. One possibility is that the default cleavage site for the R1 endonuclease is downstream of the insertion site, but the 5' end of the full-length R1 transcripts acts as a signal directing the cleavage site to the position at -9.

## Conclusions

### Origin of nascent lineages

The availability of whole genome shotgun sequences has enabled us to evaluate the level of sequence variation of the R1 and R2 elements in 12 species of *Drosophila*. The level of nucleotide divergence for most copies was typically less than 0.2%, suggesting either the elements are subject to the same concerted evolution mechanisms that enable the rRNA genes themselves to remain nearly identical, or that the R1 and R2 elements are gained and lost rapidly from the locus (that is, all copies are recent insertions). All previous analysis suggested the latter explanation. Analyses of the 5' truncated copies of R1 and R2 in several species have suggested that these elements do turnover rapidly. Different animals from the same population were found to have different collections of 5' truncated copies [44,45] and most 5' truncated elements within an animal had not undergone duplication by recombination [46,47]. Thus, the individual copies of R1 and R2 do not appear to remain in the rDNA locus for long enough periods to be substantially influenced by the recombinations leading to the concerted evolution of the locus.

One puzzling aspect concerning the evolution of R1 and R2 was the presence of multiple families in some species, yet no evidence for the origin of these lineages by horizontal transfer [28,48]. The rapid turnover of individual R1 and R2 elements suggests that each active copy can generate its own lineage, which over time should accumulate sequence variation. Thus, while separate lineages of R1 and R2 should be able to arise, the question remained as to whether they could be maintained within a species. In this report we have for the first time detected these nascent lineages of R1 and R2 elements within a species. Two distinct subfamilies of R1B elements were detected in *D. grimshawi *and two distinct subfamilies of R2 elements in *D. willistoni *(Figure 4). In addition to these distinct lineages, other species contained individual copies of R1 or R2 that had from 1% to 7% nucleotide divergence from the majority of elements in the species. Many of these divergent copies had intact ORFs, and thus were potentially active. Another frequent finding was the presence of distinct 5' UTRs for the R1 elements (Table 1). This was most widespread in *D. ananassae *where the R1A elements could be divided into two groups that diverged 10% in their 5' UTR sequences, while the R1B elements could be divided into four groups with 5' UTRs that diverged from 15% to 22%. We suggest this accelerated divergence of the 5' UTRs represents the evolution of new promoter sequences driving the transcription of the elements. Once a new promoter is formed, copies containing this new promoter may differ in their expression, thus giving rise to new lineages of elements.

Similar examples of the rapid evolution of the 5' UTRs of R2s were not detected. We have previously suggested that R2 elements do not contain their own promoter but are co-transcribed with the 28S gene [32,33]. This co-transcription may make it more difficult for independent R2 lineages to evolve. A single lineage of R2 elements has been found in the *Drosophila *genus [25], while four distinct lineages of *Drosophila *R1 elements exist [28]. It is also possible that the more frequent loss of R2 from a *Drosophila *species (3 out of 12 species) compared to R1 (no losses among the 12 species) could also be the result of R2's reliance upon co-transcription with the rDNA units in which they reside.

### Second-strand DNA synthesis

Many of the initial steps involved in the cleavage of the target site bottom strand and its use to prime reverse transcription have been characterized using purified R2 protein *in vitro *[10,38,49]. The recent discoveries that sequences near the 5' end of the RNA transcript regulate top-strand cleavage [11], and that the R2 polymerase can efficiently displace an RNA strand while using a DNA strand as template [50], suggest that the R2 protein also synthesizes the second DNA strand. However, many questions remain concerning whether and how top-strand cleavage of the target DNA is used to prime second-strand synthesis.

Because of an unusual template jumping ability of the R2 polymerase [39], we previously suggested that during reverse transcription the R2 polymerase jumps from the R2 transcript onto the upstream DNA target sequences [5,36,37]. The hundreds of R2 5' junctions analyzed here suggest a different model. In *Drosophila *we propose that the 5' ends of the R2 RNA transcripts contain terminal G residues that, after reverse transcription and top-strand cleavage, enable the terminal C residues to anneal to the G residues of the top DNA strand after cleavage (Figure 5c). This model would explain the most common full-length R2 insertions in all *Drosophila *species. Similarly, microhomologies between internal R2 sequences and the upstream 28S gene sequences can explain the priming of second-strand DNA synthesis in many 5' truncated R2 insertions. When annealing of microhomologies does not occur, non-templated residues can be observed that were either added during first-strand synthesis when the R2 polymerase runs off an RNA template [39] or during second-strand synthesis before the R2 polymerase engages the cDNA [38].

The only aspect of this integration model that does not agree with previous biochemical studies is the location of the top-strand cleavage site. The experiments using the *B. mori *R2 protein suggested this cleavage was two nucleotides upstream of the bottom-strand site [10] while our analysis of *Drosophila *R2 junctions suggest it is opposite the bottom-strand site (Figure 5c). We suggest this represents an evolved difference between the R2 elements in these divergent insects. We have analyzed the 5' junctions of over 40 *B. mori *R2 insertions and found their structure is consistent with the location of top-strand cleavage determined with the purified protein [37]. We suggest that cleavage of the top strand by the R2 endonuclease is not rigidly determined, and thus its location can vary.

Much less was previously known about the R1 retrotransposition mechanism. *In vitro *studies of the R1 endonuclease, again from *B. mori*, revealed a bottom-strand cleavage site in a position consistent with its use to prime reverse transcription [12,13]. All *Drosophila *R1 3' junctions, as well as 3' junctions in other insect species [19], are consistent with this cleavage site. As in the case of R2, the R1 5' junctions suggest cleavage of the top strand is less precise and subject to evolutionary changes. For many *Drosophila *R1 insertions top-strand cleavage was proposed to be at a site 14 bp downstream of the bottom strand, again consistent with the biochemical studies. Use of this site to prime second-strand synthesis results in a 14 bp TSD. Minor changes in this cleavage location were found in *D. ananassae*, where the R1B insertions have 11 or 13 bp TSDs, and in *D. willistoni*, where some ancestral type R1A insertions have an 8 bp TSD. Annealing of cDNA sequences to the upstream target site presumably does not occur as significant nucleotide variation is observed at the junctions.

A radical change in top-strand cleavage site preference is proposed for the R1A elements of the melanogaster species group. In these species, many R1 insertions result in a 9 bp target site deletion, suggesting cleavage frequently occurs 9 bp upstream of the bottom-strand site (Figure 10). Associated with this remarkable change in cleavage site preference also appears to be a change in the nature of the R1 RNA transcript used for retrotransposition. Based on the structure of the ISR full-length insertions and of non-28S insertions, these melanogaster-type R1 transcripts contain from 6 to 27 nucleotides of 28S sequence ending 9 bp upstream of the integration site. These upstream 28S sequences on full length R1 transcripts appear to anneal to the target site, giving rise to highly precise 5' junctions. Interestingly, these melanogaster-type R1s retain the ability to cleave downstream of the integration site, resulting in the ISRs seen in some full-length insertions and the TSDs observed for all 5' truncated insertions. Because annealing of the upstream 28S sequences is not possible with these downstream cleavage sites, significant variation is observed at these junctions. We searched for evidence of these additional 28S sequence tags among available expressed sequence tag sequences from *D. melanogaster*. However, all R1 sequences contained extensive upstream 28S sequences or were transcripts of R1 tandem arrays (data not shown).

Thus, the integration mechanisms used by the R1 and R2 elements are quite similar. Cleavage of the bottom strand and its use to prime first-strand DNA synthesis is rigidly determined, and no variation was observed between species. Cleavage of the top strand and its use to prime second-strand synthesis is flexible, which results in different junctions both within and between species. R1 and R2 are highly successful in the 28S niche. Whether change in the location of top-strand cleavage occurs simply because it is neutral or because it increases insertion efficiency is unclear. Nevertheless, the ability of R1 and R2 to explore the top-strand cleavage site suggests that variations of the retrotransposition mechanism can evolve among arthropods that could affect both the elements and the rDNA loci in which they reside.

## Materials and methods

### Species and databases

Original sequencing reads from the whole genome shotgun sequencing projects were accessed by Blast search (version 2.2.17) in the trace archives at NCBI [51]. The 12 *Drosophila *species and their sequencing fold coverage were: *D. ananassae *(9-fold), *D. erecta *(10-fold), *D. grimshawi *(8-fold), *D. melanogaster *(12-fold), *D. mojavensis *(8-fold), *D. persimilis *(4-fold), *D. pseudoobscura *(9-fold), *D. sechellia *(5-fold), *D. simulans *(4-fold; white 501 strain only), *D. virilis *(8-fold), *D. willistoni *(8.4-fold) and *D. yakuba *(9-fold).

### Assembling R1 and R2 elements

Downstream 28S sequences flanking the known R1 and R2 insertion sites were used as Blast queries using default parameters except for requesting the maximum of 20,000 target sequences. The reads were collected, trimmed upstream of the query and aligned in ClustalX [52]. Those sequences upstream of the query that were not 28S rRNA sequences were considered putative R1 or R2 elements and were used as Blast queries in the nucleotide collection database (nr/nt) and VecScreen at NCBI [51]. Some putative elements were identified as cloning vector sequence and disregarded. For the remaining majority of sequences, 5' extensions of the sequences were assembled by Blast search, followed by alignment and extraction of a consensus. Iterations were done until the 5' junction of the element with the 28S was reached. Full assemblies were not possible in a few instances because of low element copy number and low coverage of some genome sequences.

### Phylogenetics

Alignments of concatenated 18S and 28S sequences (from Stage and Eickbush [18] and available at the Eickbush lab website [53]) were done in ClustalX [52] with minor manipulations done in Jalview [54]. Default parameters of phyML version 3.0 [55] as implemented through the LIRMM website [56,57] were used to construct maximum likelihood trees and visualized using TreeDyn 198 [58]. Branch support values were the minimum score based on Shimodaira-Hasegawa-like and Chi2-based approximate likelihood-ratio tests [59].

### Nucleotide variation in the 5' and 3' ends of elements and sequence divergence between species

Blast queries were 75 bp long near the 5' or 3' end of each consensus R1 or R2 sequence; default Blast parameters were used except for requesting the maximum of 20,000 target sequences and using a match/mismatch score of 1,-1. The Blast results were trimmed to include 400 bp of the element and 100 bp of flanking sequence. Retention of the flanking sequence enabled those elements present in the 28S gene to be separated from those copies inserted elsewhere in the genome. ClustalX alignment [52] of results from each Blast search was conducted and groups of like sequences were extracted and consensus sequences derived. Quality scores for reads containing differences from the main consensus were examined to verify that the variation observed was not sequencing error (scores less than 40 were considered sequencing error). The Sequence Manipulation Suite: Ident and Sim [60] aided determination of the percent identity of the variants relative to the consensus.

In theory it should be possible to make copy number estimates based on the genome sequence coverage and the number of trace reads recovered. In practice, however, random variation in fold coverage for any given sequence means only an estimate of copy number for repetitive sequences can be inferred. In addition, rDNA loci in many species of *Drosophila *are located on the X and Y chromosomes, resulting in different sequencing coverage. Finally, by requiring analyzed reads to cover 400 bp to enable a significant and uniform level of information to be recovered from each read, fewer reads were obtained for each analysis. Thus, estimates of element abundance were made relative to the number of rDNA units also recovered in the trace archives (that is, fraction of rDNA units inserted). Copies with small numbers of reads (approximately 2 to 12) and identical sequence variation were interpreted as single elements.

### Insertions outside the 28S genes

Using the 5' and 3' ends of R1 and R2 elements as Blast queries and analyzing the flanking sequences, we found many examples of R1 elements inserted outside the usual 28S site. Identification of the non-28S sequences was attempted by comparing them to our assembled rDNA-related sequences and by Blast search of the NCBI non-redundant nucleotide database [51]. While a few target sites could be identified, the majority of sequences had not been characterized and represented repeated sequences, and thus were presumed to be heterochromatic. We then attempted to find uninserted copies of the sequences using the flanking sequences as Blast queries. In those cases where the target site was repeated, we could identify examples of uninserted target sites, and in some instances the opposite junction of the R1 insertion.

### dN/dS analysis

Selection analysis was done using the HyPhy package available at Datamonkey [61]. We conducted all the available tests and report here the dN/dS results of the PARRIS test [62], which were similar in all the tests.

## Abbreviations

ISR: insertion site rearrangement; ITS: internal transcribed spacer; LTR: long terminal repeat; ORF: open reading frame; rDNA loci: loci encoding the tandem array of rRNA genes; TE: transposable element; TPRT: target primed reverse transcription; TSD: target site duplication; UTR: untranslated region.

## Authors' contributions

DS participated in the design, carried out all the experiments and drafted the manuscript. TE participated in the design and helped with the manuscript. Both authors read and approved the final manuscript.

## Additional data files

The following additional data are available with the online version of this paper. Each file contains the consensus nucleotide sequence for an R1 or R2 element: *Drosophila ananassae *R2 (Additional data file 1); *Drosophila melanogaster *R2 (Additional data file 2); *Drosophila persimilis *R2 (Additional data file 3); *Drosophila pseudoobscura *R2 (Additional data file 4); *Drosophila sechellia *R2 (Additional data file 5); *Drosophila simulans *R2 (Additional data file 6); *Drosophila willistoni *R2.1 (Additional data file 7); *Drosophila yakuba *R2 (Additional data file 8); *Drosophila ananassae *R1A (Additional data file 9); *Drosophila ananassae *R1B (Additional data file 10); *Drosophila erecta *R1 (Additional data file 11); *Drosophila grimshawi *R1.1 (Additional data file 12); *Drosophila mojavensis *R1 (Additional data file 13); *Drosophila melanogaster *R1 (Additional data file 14); *Drosophila persimilis *R1 (Additional data file 15); *Drosophila pseudoobscura *R1 (Additional data file 16); *Drosophila sechellia *R1 (Additional data file 17); *Drosophila simulans *R1 (Additional data file 18); *Drosophila virilis *R1 (Additional data file 19); *Drosophila willistoni *R1 (Additional data file 20); *Drosophila yakuba *R1 (Additional data file 21); *Drosophila grimshawi *R1.2 (Additional data file 22); *Drosophila willistoni *R2.2 (Additional data file 23).

## Supplementary Material

Additional data file 1 *Drosophila ananassae *R2 complete consensus sequence. Click here for file

Additional data file 2 *Drosophila melanogaster *R2 complete consensus sequence. Click here for file

Additional data file 3 *Drosophila persimilis *R2 complete consensus sequence. Click here for file

Additional data file 4 *Drosophila pseudoobscura *R2 complete consensus sequence. Click here for file

Additional data file 5 *Drosophila sechellia *R2 complete consensus sequence. Click here for file

Additional data file 6 *Drosophila simulans *R2 complete consensus sequence. Click here for file

Additional data file 7 *Drosophila willistoni *R2 complete consensus sequence. Click here for file

Additional data file 8 *Drosophila yakuba *R2 complete consensus sequence. Click here for file

Additional data file 9 *Drosophila ananassae *R1A complete consensus sequence. Click here for file

Additional data file 10 *Drosophila ananassae *R1B complete consensus sequence. Click here for file

Additional data file 11 *Drosophila erecta *R1 complete consensus sequence. Click here for file

Additional data file 12 *Drosophila grimshawi *R1 complete consensus sequence. Click here for file

Additional data file 13 *Drosophila mojavensis *R1 complete consensus sequence. Click here for file

Additional data file 14 *Drosophila melanogaster *R1 complete consensus sequence. Click here for file

Additional data file 15 *Drosophila persimilis *R1 complete consensus sequence. Click here for file

Additional data file 16 *Drosophila pseudoobscura *R1 complete consensus sequence. Click here for file

Additional data file 17 *Drosophila sechellia *R1 complete consensus sequence. Click here for file

Additional data file 18 *Drosophila simulans *R1 complete consensus sequence. Click here for file

Additional data file 19 *Drosophila virilis *R1 complete consensus sequence. Click here for file

Additional data file 20 *Drosophila willistoni *R1 complete consensussequence. Click here for file

Additional data file 21 *Drosophila yakuba *R1 complete sequence. Click here for file

Additional data file 22 *Drosophila grimshawi *R1.2 complete consensus sequence. Click here for file

Additional data file 23 *Drosophila willistoni *R2.2 complete consensus sequence. Click here for file
